# Fracture Risk Assessment in Chronic Kidney Disease, Prospective Testing Under Real World Environments (FRACTURE): a prospective study

**DOI:** 10.1186/1471-2369-11-17

**Published:** 2010-08-20

**Authors:** Sarah L West, Charmaine E Lok, Sophie A Jamal

**Affiliations:** 1 Multidisciplinary Osteoporosis Program, Women's College Hospital, and Department of Exercise Sciences, University of Toronto, Toronto, Ontario, Canada; 2 Toronto General Hospital, and University of Toronto, Toronto, Ontario, Canada; 3 Multidisciplinary Osteoporosis Program, Women's College Hospital, and Division of Endocrinology and Metabolism, University of Toronto, Toronto, Ontario, Canada

## Abstract

**Background:**

Chronic kidney disease (CKD) is associated with an increased risk of fracture. Decreased bone mass and disruption of microarchitecture occur early in the course of CKD and worsens with the progressive decline in renal function so that at the time of initiation of dialysis at least 50% of patients have had a fracture. Despite the excess fracture risk, and the associated increases in morbidity and mortality, little is known about the factors that are associated with an increase in fracture risk. Our study aims to identify prognostic factors for bone loss and fractures in patients with stages 3 to 5 CKD.

**Methods:**

This prospective study aims to enroll two hundred and sixty men and women with stages 3 to 5 CKD. Subjects will be followed for 24 months and we will examine the ability of: 1) bone mineral density by dual x-ray absorptiometry at the spine, hip, and radius; 2) volumetric bone density by high resolution peripheral quantitated computed tomography at the radius and tibia; 3) serum markers of bone turnover; 4) bone formation rate by bone biopsy; and 5) muscle strength and balance to predict spine and non-spine fractures, identified by self-report and/or vertebral morphometry. All measurements will be obtained at baseline, at 12 and at 24 months with the exception of bone biopsy, which will be measured once at 12 months. Subjects will be contacted every 4 months to determine if there have been incident fractures or falls.

**Discussion:**

This study is one of the first that aims to identify risk factors for fracture in early stage CKD patients. Ultimately, by identifying risk factors for fracture and targeting treatments in this group-before the initiation of renal replacement therapy - we will reduce the burden of disease due to fractures among patients with CKD.

## Background

Chronic kidney disease (CKD) affects 5-10% of the world population [1] and is associated with many adverse outcomes including bone disorders and fractures. Osteoporosis is characterized by a reduction in bone mass and disruption of skeletal microarchitecture leading to an increased susceptibility to fracture with minimal trauma. Decreased bone mass and disruption of microarchitecture occur early in the course of CKD and worsens with the progressive decline in renal function so that at the time of initiation of dialysis at least 50% of patients have had a fracture [2-5]. After a hip fracture, the average length of stay in an acute care hospital is 3 weeks; 25% of patients must remain in long-term care institutions for at least one year, and a third who return home must depend on other people or devices for mobility [6].

**Table 1:** Stages of Chronic Kidney Disease [14].

Stage	Description	**Glomerular Filtration Rate (GFR); mL/min/1.73 m**^**2**^
1	Kidney damage with normal or increased GFR	≥90
2	Kidney damage with mild decrease in GFR	60 to 89
3	Moderate decrease in GFR	30 to 59
4	Severe decrease in GFR	15 to 29
5	Kidney Failure	<15 or dialysis

The prevalence of osteoporotic hip and vertebral fractures among dialysis patients exceeds that of the general population, with profound effects on morbidity and mortality [4,5,7-11]. A recent retrospective study [12] reported that the incidence of hip fractures in a hemodialysis population was 17.4 times greater and occurred at a younger age compared to the general population (16 and 13 years younger among men and women, respectively). Furthermore, patients with CKD stage 5 who require renal replacement therapy (also known as end stage renal disease (ESRD)) had a one-year mortality rate of 64% following a hip fracture compared with 15 to 20% in the general population.

The first step to decreasing the morbidity and mortality associated with fractures in patients with CKD is to identify those patients at high risk, in order to direct appropriate preventative strategies. While most research has focused on identifying prognostic factors for fracture among ESRD patients, it excludes the earlier stages of CKD and also has several pragmatic limitations. For example, dialysis patients are frequently reluctant to participate in studies due to the required additional time commitments outside of regularly scheduled dialysis sessions. As well, assessments may be invasive, and may not have direct patient benefit. As a result, most bone related studies in ESRD involve a select population comprising small numbers of relatively young, healthy patients, with limited clinical assessments [7,8,13-18]. This limits the generalizability of these studies. Finally, because of differences in mineral metabolism with decreasing renal function, the risk factors for fracture in patients with ESRD may not be the same as the risk factors for fracture in patients with earlier stages of CKD.

The etiology of fractures in patients with CKD is multifactorial. Factors that may play a role include decreases in the quantity of bone or osteoporosis which can be identified by bone mineral density (BMD) testing, and alterations in the quality of bone. Bone quality can be indirectly assessed by markers of bone turnover and peripheral quantitated computed tomography (pQCT) and directly assessed with bone biopsy. Other risk factors for fracture in patients with CKD may be poor nutrition, inactivity, and an increased risk of falling due to myopathy and peripheral neuropathy [19]. As a result, there are no recommendations or standard methods of practice to assess fracture risk. Indeed most clinicians caring for patients with CKD do not utilize BMD testing, measurement of bone turnover markers, pQCT or bone biopsy to assess fracture risk. Bone biopsy, in particular, is not used in clinical practice to assess fracture risk. Further, those clinicians who do order the occasional noninvasive test to assess fracture risk in patients with CKD may not know how to confidently interpret the results. The FRACTURE study (**F**racture **R**isk **A**ssessment in **C**hronic Kidney Disease, Prospective **T**esting **U**nder **R**eal World **E**nvironments) has been designed to expand the current knowledge of prognostic factors for fracture in ESRD to the CKD stage 3 to 5 population and address some of the existing knowledge gaps in the identification, diagnosis, and interpretation of bone disease and fracture risk in CKD.

### Study Aim

The aim of this study is to prospectively identify prognostic factors for bone loss and fractures in patients with stages 3 to 5 CKD.

### Study Hypotheses

We will test four primary hypotheses in patients with CKD stages 3-5:

1. BMD at cortical sites (the mid radius) will be associated with fractures.

2. Volumetric BMD at the forearm by High Resolution-pQCT (HR-pQCT) will be associated with fractures.

3. There will be a positive correlation between the neuromuscular testing (i.e., the timed up and go) and presence of fractures.

4. There will be a positive association between fractures and the presence of either: adynamic bone disease, or hyperparathyroid bone disease, as defined by the bone formation rate (BFR).

## Methods

### Study Design and Setting

This is a 24-month prospective cohort study of 260 men and women attending outpatient renal clinics at the University Health Network (UHN), and at St. Michael's Hospital in Toronto, Ontario, Canada. Other sites will be recruited as necessary. Subjects will undergo all testing (described below) upon study entry and then at 12 and 24 months with the exception of bone biopsy, which will be performed only once at 12 months. Subjects who progress to ESRD will have assessments at the time of dialysis initiation or transplantation, and then 12 months later. Subjects will also be contacted by telephone every 4 months, at which time they will be asked about the occurrence of falls and fractures since their last study contact.

### Ethical Considerations

This study has been reviewed by the local Research Ethics Boards, and has received ethics approval at all participating institutions.

### Eligibility

Inclusion Criteria: Men and women aged 18 years and older, with a glomerular filtration rate of ≤60 mL/min/1.73 m^2 ^at study entry are eligible for recruitment.

Exclusion Criteria: Patients who are unable to give informed consent, unable to have spinal radiographs or BMD measurements; those taking bisphosphonates, calcitonin, hormone replacement therapy, or the oral contraceptive pill are excluded. Subjects allergic to tetracycline are excluded from the bone biopsy component of the study.

### Predictor Variables

All assessments (below), with the exception of bone biopsy, will be obtained at a single site (UHN) using standard protocols.

#### i. Bone Mineral Density

BMD will be measured at the lumbar spine (L1 to L4), the total hip, femoral neck and proximal radius using a bone densitometer (Hologic). To reduce variability, BMD will be measured using a standard protocol (including regular use of a phantom spine). BMD tests will be reported according to the International Society for Clinical Densitometry (ISCD) protocol by a single ISCD certified clinician blinded to the study hypotheses.

#### ii. High Resolution Peripheral Quantitative Computed Tomography

HR-pQCT measurements will be obtained at the non-dominant radius (non-weight bearing site), and also at the tibia (weight bearing site) using the Xtreme CT device (Scanco Medical AG, Basserdorf, Switzerland). Measurements that can be obtained from HR-pQCT include: total volumetric density, cortical volumetric density, trabecular volumetric density, cortical thickness, trabecular thickness, trabecular separation, and trabecular number. The reproducibility of density measurements is <0.3% CV (phantom) [20].

#### iii. Markers of Bone Turnover

Blood samples will be collected, and then centrifuged at 4°C for 15 minutes. Serum will be aliquotted and immediately stored in microtubes at -80°C for future measurement of bone turnover markers and bone cytokines.

#### iv. Bone biopsy

Bone histomorphometry will be performed in a subgroup of 50 subjects. To ensure adequate power, 25 subjects with confirmed prevalent fractures will be included, as well as 25 without confirmed fracture. We will obtain a bone biopsy of the anterior superior iliac crest at 12 month follow up only.

Tetracycline labeling:

Subjects will be given tetracycline labels administered over ~2 week period as follows: Two days of tetracycline (250 mg, by mouth, four times per day), and then given a 12 day intermission (days 3-14). On day 15, subjects will be given the same tetracycline (250 mg, by mouth, four times per day) for 4 days (days 15-18). Anterior iliac crest bone biopsies will be obtained one to two days later. By labeling twice with tetracycline standard histomorphometry can determine the mineral apposition rate and mineralizing perimeter for the two separate labeling periods and thereby calculate the BFR.

Bone biopsy technique:

The iliac biopsy will be obtained following the protocol of Meunier [21], using a Bordier trephine, 8 mm in internal diameter, to avoid fracture and compression of the core of bone and minimize sampling error. The procedure is performed under local anesthesia, supplemented by parenteral sedation if needed to achieve proper muscle relaxation.

Samples will be processed at 4°C according to standardized techniques. The primary histomorphometric variable to be studied is the BFR. The classification of bone disease associated with ESRD is based on the BFR and it has been purported that either an increased BFR (e.g. >500 μm/mm tissue area/day; as seen in hyperparathyroidism) or a decreased BFR (e.g. <108 μm/mm tissue area/day; as seen in adynamic bone disease), may be the strongest predictor of fractures. We will determine if the BFR is a prognostic factor for fracture by first classifying turnover into low, normal or high, based on the reference values noted above.

#### v. Muscle Strength, Balance and Falls

Muscle strength and balance will be assessed using three tests: The Timed Up and Go (TUG), Hand Grip, and in a subgroup of subjects, the 6-Minute Walk Test (6MW). These tests allow determination of muscle strength and balance across a wide range of functional abilities, has been validated in populations with renal disease and has been shown to be associated with fractures in cross sectional studies of patients with ESRD [3].

### Primary Outcome Variable

#### i. Spine and Nonspine Fractures

Subjects will be questioned about a history of non-spine fractures at baseline and every 4 months during the follow up. Fractures will be classified as either traumatic or "low trauma" (osteoporotic) based on World Health Organization criteria; an osteoporotic fracture is defined as a fracture from one's standing height or less. Data from a prospective study demonstrate that self-report of fractures is accurate for osteoporotic fractures [22]. Subjects will have anterior and lateral radiographs of the thoracic and lumbar spine at study entry to evaluate the presence of baseline (prevalent) vertebral fractures and then at 12 and 24 months to assess the development of new (incident) vertebral fractures. All radiographs will be obtained and reviewed using a standardized methodology, vertebral morphometry [23], by a muscular-skeletal radiologist with expertise in the radiographic diagnosis of vertebral fractures.

### Withdrawal from Study

If requested, subjects will be withdrawn from the study without prejudice, as documented and explained at the time of consenting.

### Statistical Considerations

#### i. Sample Size Estimations

Based on our prior experience with recruiting subjects with ESRD, and the natural history of CKD the study anticipates recruitment of 260 subjects over 2 years. It is estimated that of these 260 subjects, 60 will have renal transplants, progress to ESRD or die, leaving 200 subjects. The power calculations were based on comparisons of mean baseline values between 30% of patients with prevalent fractures and 70% without prevalent fractures. This 30% is a conservative estimate given prior data that demonstrates that 50% of CKD patients have sustained a fracture prior to starting dialysis [2-5], and data from the Canadian Multicentre Osteoporosis Study demonstrates that among a population based sample of healthy men and women 50 years and older 38% of men and 41% of women have had a fracture [24]. A sample size of 200 subjects is adequate to meet all our hypotheses. Sample size calculations for each of the primary hypotheses are detailed below:

##### 1. BMD at cortical sites (the mid radius) will be associated with fractures

Based on a review of the literature, a well accepted minimal clinically important difference (MCID) in BMD between fractured and non-fractured patients is 3%, which is associated with standard deviations (SD) that range from 4.5% to 6.8% [25-32]. We will have 81% (at a SD of 6.8%) to 99% power (at a SD of 4.5%) to detect a 3% difference in mid radius BMD.

##### 2. Volumetric BMD at the forearm by HR-pQCT will be associated with fractures

The limited published data suggest that an MCID in volumetric BMD ranges from 1 to 3 with a SD that varies from 1 to 2 [20]. The most conservative assumption is a MCID of 1 with an SD 2. We will have 89% power to detect this MCID.

##### 3. There will be a positive correlation between the "TUG" and presence of fractures

Our data demonstrates a MCID of 5 seconds that can be associated with a SD of 10 to 12 seconds. We will have 89 to 76% power to detect this difference.

##### 4. There will be a positive association between fractures and the presence of either: adynamic bone disease, or hyperparathyroid bone disease, as defined by the BFR

Power calculations for this hypothesis were based on the assumption that of the 60 patients with prevalent fractures (30% of 200) just under half (40%) would agree to biopsy (25 subjects) and we would recruit another 25 non-fractured subjects for a total of 50. If we assume that 85% of patients with fracture would have an abnormal BFR, we have 80% power to detect an abnormal BFR rate of 45% or lower in the non-fractured group. We anticipate that approximately 45% of subjects will have incident fractures in the two-year follow-up period.

#### ii. Statistical Analyses

We will use linear regression models to assess relationships, with BMD, volumetric BMD (by HR-pQCT), TUG and BFR (from bone biopsy) as predictor variables and presence or absence of prevalent fracture as the main outcome variable. Unadjusted analyses will be performed to determine the association between each predictor variable of interest and fracture outcome. Those with a positive association will be adjusted for potentially confounding covariates such as gender, age, weight, serum calcium, diabetes, and intact serum PTH. Goodness-of-fit will be evaluated for all models by inspecting plots of residuals, as well as use of Hosmer and Lemeshow Goodness of fit test; variables will be transformed as necessary. Our primary analyses will not be stratified by gender as earlier work by our group has not demonstrated a significant difference in fractures by gender among patients with renal disease. However supplementary analyses will be performed separately by gender and by the presence of type II diabetes. We will consider a p value of less than 0.05 statistically significant and we will not adjust for multiple comparisons. Our study statistician will perform our analyses using STATA version 10 (STATA Corp. College Stn. TX) and R 2.7 (R Development Core Team (2008). R: A language and environment for statistical computing. R Foundation for Statistical Computing, Vienna, Austria)

### Anticipated Challenges and Solutions

We have anticipated some potential challenges and have offered corresponding solutions:

#### 1. Subjects drop out after enrollment

Based on chart review of the Renal Management Clinic at the UHN, approximately 15 to 20% of subjects progress to ESRD, receive a transplant, transfer care or die every year, with very few patients lost to follow up. To account for these potential "drop outs" after enrollment, we will recruit 260 subjects, in anticipation that 200 will continue to be followed.

#### 2. Unable to recruit patients from single site

The clinic at UHN has a potential pool of over 500 subjects, however, we have established collaborative links with St. Michael's Hospital in Toronto as well as with other Toronto area institutions, which will assist us in recruiting and meeting our sample size goals.

#### 3. Failure to obtain adequate samples for bone biopsy component

It is possible, due to the invasive nature of the bone biopsy procedure that few patients will consent. To maximize the number of biopsies and to limit drop outs, the protocol was designed so that we obtain a single bone biopsy at one year follow up, rather than obtaining repeat bone biopsy samples. Note that there is a paucity of data on bone histomorphometry and stages of CKD - thus at a minimum our findings will serve as pilot data for a larger study.

## Discussion

This study is designed to identify risk factors for fracture in early stage CKD patients. To our knowledge, no other prospective studies in Canada have been conducted to determine fracture risk factors in patients with stage 3-5 CKD, not yet on renal replacement therapy. Ultimately, by identifying risk factors for fracture and targeting treatments in this group - before initiating renal replacement therapy - we will reduce the burden of disease due to fractures among patients with ESRD.

## Competing interests

The authors declare that they have no competing interests.

## Authors' contributions

SAJ and CL are responsible for the design of this prospective study, the construction of the protocol, manuscript review, obtaining peer reviewed funding, and data analysis. SLW is responsible for compiling this manuscript, ethics submission, and is involved with recruitment and retention. All authors read and approved the final manuscript.

## Pre-publication history

The pre-publication history for this paper can be accessed here:

http://www.biomedcentral.com/1471-2369/11/17/prepub
